# Cubic Iron Core–Shell Nanoparticles Functionalized to Obtain High-Performance MRI Contrast Agents

**DOI:** 10.3390/ma15062228

**Published:** 2022-03-17

**Authors:** Maria Volokhova, Anna Shugai, Masahiko Tsujimoto, Anna-Liisa Kubo, Sven Telliskivi, Mait Nigul, Peep Uudeküll, Heiki Vija, Olesja M. Bondarenko, Jasper Adamson, Anne Kahru, Raivo Stern, Liis Seinberg

**Affiliations:** 1 National Institute of Chemical Physics and Biophysics, Akadeemia tee 23, 12618 Tallinn, Estonia; maria.volokhova@kbfi.ee (M.V.); anna.shugai@kbfi.ee (A.S.); anna-liisa.kubo@kbfi.ee (A.-L.K.); peep.uudekull@gmail.com (P.U.); heiki.vija@kbfi.ee (H.V.); olesja.bondarenko@kbfi.ee (O.M.B.); jasper.adamson@kbfi.ee (J.A.); anne.kahru@kbfi.ee (A.K.); raivo.stern@kbfi.ee (R.S.); 2 Department of Chemistry and Biotechnology, Tallinn University of Technology, Akadeemia tee 15, 12618 Tallinn, Estonia; 3 Institute for Integrated Cell-Materials Sciences, Kyoto University, Yoshida-Ushinomiyacho, Sakyo-ku, Kyoto 606-8501, Japan; tsujimoto@icems.kyoto-u.ac.jp; 4 North Estonia Medical Centre, 19 J. Sütiste tee, 13419 Tallinn, Estonia; s.telliskivi@gmail.com; 5 The Radiology Clinic of Tartu University Hospital, 1a L. Puusepa St., 50406 Tartu, Estonia; mait.nigul@kliinikum.ee

**Keywords:** nanomaterials, synthesis, magnetic properties, MRI contrast agents, toxicity, ROS

## Abstract

Nanoparticles with SiO ${}_{2}$ coating were synthesized to have a cubic iron core. These were found to have saturation magnetization very close to the highest possible value of any iron-containing nanoparticles and the bulk iron saturation magnetization. The in vitro toxicology studies show that they are highly biocompatible and possess better MRI contrast agent potential than iron oxide NPs.

## 1. Introduction

There has been considerable interest in magnetic nanoparticles (NPs) over recent decades due to their use in pharmaceutical and biomedical applications, including drug delivery [1], hyperthermia treatment [2,3] and magnetic resonance imaging (MRI) [4,5,6]. The most developed of these fields is the use of superparamagnetic iron oxide nanoparticles (SPIONs) as T ${}_{2}$ -negative contrast-enhancing agents in MRI [5,7]. Multiple aspects, including biocompatibility, size, shape, charge and magnetic properties of the NPs, need to be taken into account when developing the most efficient NPs for use in high-performance MRI measurement [5,6,7,8]. SPIONs are efficient in lowering the transverse relaxation time (T ${}_{2}$ ) of water proton spins in tissues. This process is described by relaxivity, r ${}_{2}$ , given by the equation

$$\frac{1}{T_{2,m}}=r_{2}*C_{m}$$

where C ${}_{m}$ is the total concentration of magnetic ions [9]. The total relaxation rate is then given by the equation

$$\frac{1}{T_{2}}=\frac{1}{T_{2,0}}+\frac{1}{T_{2,m}}$$

where 1/T ${}_{2,0}$ is the relaxation rate in the absence of magnetic NPs. Accordingly, for the lowest T ${}_{2}$ relaxation time of proton spins, high r ${}_{2}$ values are desired. One downside of SPIONs is that their saturation magnetization ( $M_{s}$ ) is clearly below that of bulk iron. Typically, investigated SPIONs, magnetite (Fe ${}_{3}$ O ${}_{4}$ ) and its oxidized form maghemite ( γ -Fe ${}_{2}$ O ${}_{3}$ ), have $M_{s}$ values of 124 emu per g-Fe and 109 emu per g-Fe, respectively [10]. Metallic iron’s ( α -Fe) saturation magnetization of 218 emu per g-Fe exceeds these numbers nearly twofold. From the perspective of MRI, high $M_{s}$ values are crucial for MRI signal enhancement since contrast agents with high $M_{s}$ increase the relaxation rate of proton spins according to 1/T ${}_{2},m$ *∞* $M_{s}$ ${}^{2}$ [11]. Therefore, based on increased $M_{s}$ , metallic α -Fe NPs have potential to produce better quality images for high-performance MRI [8]. The latter argument has prompted this study, where we synthesize and investigate NPs with a metallic cubic iron core. Solution-based synthesis of spherical α -Fe NPs with cPEG coating was performed by the optimized chemical reduction of ferrous chloride with sodium borohydride [3]. Another study on PEGylated Fe@Fe ${}_{3}$ O ${}_{4}$ NPs showed that they are a promising MRI contrast agent [12]. Additionally, iron NPs with TRITC–dextran coating (size <20 nm) were synthesized using cryomill and with saturation magnetization (180 per g-Fe) [13].

Herein, we focus on solid state synthesis of α -Fe NPs and their MRI relaxivity measurements. Traditionally, in solid state, the preparation method for spherical SiO ${}_{2}$ -coated α -Fe NPs is carried out via reduction of spherical SiO ${}_{2}$ -coated iron oxide NPs with H ${}_{2}$ gas under high temperature [14,15]. Recently, Yamamoto et al. used CaH ${}_{2}$ as a reducing agent for the synthesis of spherical α -Fe NPs and lowered the reduction working temperature down to 20–300 ${}^{\circ }$ C [16]. They later demonstrated this reduction method to be applicable for spherical α -Fe@SiO ${}_{2}$ NP synthesis from spherical Fe ${}_{3}$ O ${}_{4}$ @SiO ${}_{2}$ NPs [8,17]. Furthermore, saturation magnetization of the spherical SiO ${}_{2}$ -coated Fe nanoparticles increased with decreasing SiO ${}_{2}$ thickness [17]. Herein, we apply this reduction method on cubic core α -Fe ${}_{2}$ O ${}_{3}$ @SiO ${}_{2}$ to obtain α -Fe@SiO ${}_{2}$ NPs with an unusual cubic core morphology and furthermore characterize their magnetic and relaxation properties on a clinical 3.0 T MRI instrument. Cubic shape is important due to the change in the magnetic properties and lower saturation magnetization could expand the application to T1 or a dual mode contrast agent. For biomedical applications, NPs with biocompatible shells, e.g., coated by inert inorganic materials (such as SiO ${}_{2}$ shell) or various hydrophilic polymers (such as albumin, polyethylene glycol) are desirable [6,18]. Coating α -Fe NPs has a further advantage of stabilizing the NPs against oxidation as their magnetic properties are altered when oxidation occurs. Further, coating of the NPs with silane moieties has shown improvement of NP colloidal stability and additionally provides ample opportunities to decorate the NP surface with functional molecules [8].

Moreover, an NH ${}_{2}$ -silane coating can render the NPs dispersible in aqueous solutions over a wide pH range [19], link to biomolecules, including applications in DNA and RNA purification [20] and enhance cellular uptake of the NPs without increased cytotoxicity [21].

The generation of reactive oxygen species (ROS) has been proposed as the main mechanism behind the adverse effects of iron oxide NPs [18,22]. Uncoated iron oxide NPs are usually significantly more toxic than coated NPs because Fe ions are efficient inducers of ROS production. In addition, the surface of NPs can contribute into catalytic ROS production by amplifying the role of chemical reactions occurring at the surface [23], while the coating functions as a barrier to reduce the toxicity [24]. While some authors reported no or low toxicity even for uncoated iron oxide NPs to various human cell lines and primary human cells in vitro [25,26,27], others demonstrated moderate toxicity, e.g., toxicity at exposure concentrations below 100 mg/L [22,28].

**Scheme 1:**
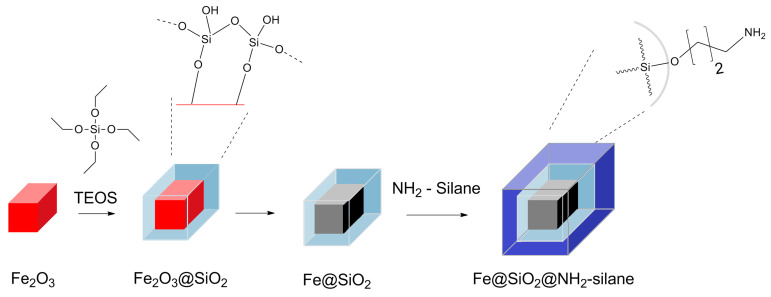
SiO ${}_{2}$ -coated α -Fe@SiO ${}_{2}$ cubic NP synthesis schema and their further functionalization.

Firstly, we report the synthesis of SiO ${}_{2}$ -coated iron metal core ( α -Fe@SiO ${}_{2}$ ) NPs with a cubic morphology (Scheme 1). Secondly, we characterize the cubic core/shell NPs’ magnetic properties and show that they possess enhanced MRI relaxivity as compared to their iron oxide counterparts ( γ -Fe ${}_{2}$ O ${}_{3}$ @SiO ${}_{2}$ ). Thirdly, we functionalize the α -Fe@SiO ${}_{2}$ NPs with 3-aminopropyltrimethoxysilane ( α -Fe@SiO ${}_{2}$ @NH ${}_{2}$ -silane) and, finally, perform nanotoxicology studies to demonstrate their low cytotoxicity.

## 2. Results and Discussion

**Scheme 2:**
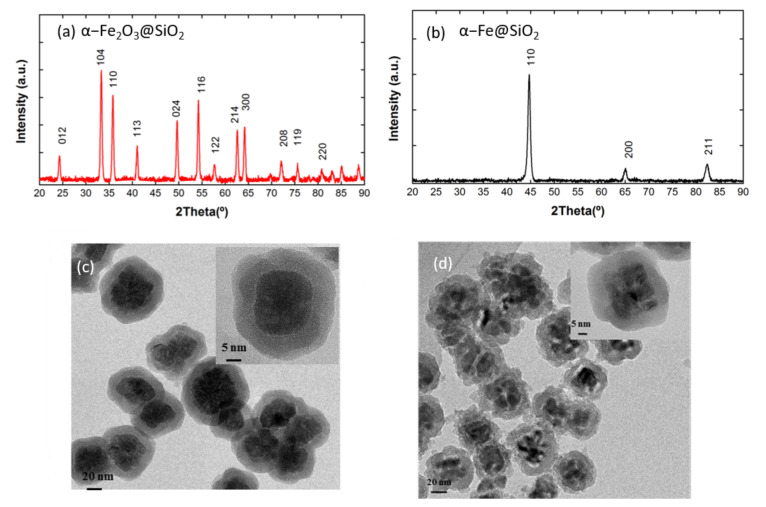
(**a**) PXRD patterns of α -Fe ${}_{2}$ O ${}_{3}$ @SiO ${}_{2}$ and (**b**) α -Fe@SiO ${}_{2}$ after CaH ${}_{2}$ reduction reaction of α -Fe ${}_{2}$ O ${}_{3}$ @SiO ${}_{2}$ . (**c**) TEM images obtained from α -Fe ${}_{2}$ O ${}_{3}$ @SiO ${}_{2}$ nanocubes and (**d**) from α -Fe@SiO ${}_{2}$ nanocubes.

Described below are experimental details, which have been limited to the requisite minimum. Further details are presented in Supplementary Materials (SM). Monodispersed cubic α -Fe ${}_{2}$ O ${}_{3}$ NPs were synthesized by a facile one-step solvothermal route from ferric nitrite [Fe(NO ${}_{3}$ ) ${}_{3}$ *9H ${}_{2}$ O], N,N-dimethyl formamide (DMF) and poly(N-vinyl-2-pyrrolidone) up to 200 ${}^{\circ }$ C [29,30]. The crystallographic structure of the NPs was confirmed by powder X-ray diffraction (PXRD) analysis, shown in Scheme 2a. The PXRD pattern revealed the hematite phase of iron oxide with characteristic reflections at 24.1 ${}^{\circ }$ , 33.2 ${}^{\circ }$ , 35.6 ${}^{\circ }$ , 40.8 ${}^{\circ }$ , 49.5 ${}^{\circ }$ , 54.1 ${}^{\circ }$ , 62.5 ${}^{\circ }$ and 64.0 ${}^{\circ }$ with the Miller indexes closest matching peak locations of hematite iron oxide phase PDF card 033-0664 from the ICDD PDF-2 database. The NPs with a cubic core shape and with the cube’s edge were observed to be 40 nm using transmission electron microscope (TEM) analysis (Scheme 2c). The NPs were then coated with a SiO ${}_{2}$ layer via a modification of the method published by Yamamoto et al. TEM images confirm the SiO ${}_{2}$ coating thickness to be 10 nm on average (Scheme 2c).

**Scheme 3:**
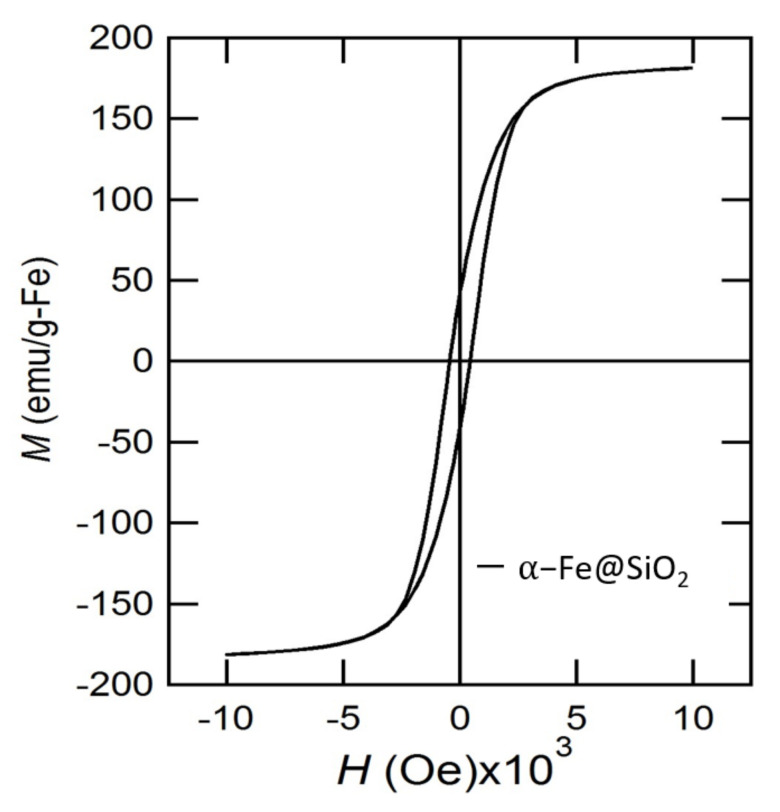
Magnetic hysteresis curve of cubic core α -Fe@SiO ${}_{2}$ showing the saturation magnetization of 180 emu/g-Fe.

Subsequently, the cubic core shape α -Fe ${}_{2}$ O ${}_{3}$ @SiO ${}_{2}$ NPs were subject to reduction with CaH ${}_{2}$ to obtain SiO ${}_{2}$ -coated α -Fe@SiO ${}_{2}$ NPs (Scheme 2d). The reduction reaction was a modification of the procedure described by Yamamoto recently [16]. The NP and CaH ${}_{2}$ mixture was heated at 260 ${}^{\circ }$ C for 4 days (for a detailed description, see the SM). The color of the reaction mixture changed from orange-red to black, indicating the formation of metal iron Fe0. The structure of the pure metallic body-centered-cubic (bcc) α -Fe core was confirmed by PXRD analysis (Scheme 2b) with 110, 200 and 211 peaks indexed [16]. TEM images in Scheme 2d reveal voids in the reduced NPs due to oxygen leaving the iron oxide NPs upon reduction. Therefore, the morphology of the as-synthesized NPs could be referred to as quasi- or pseudo-cubic core. We then characterized the NPs’ magnetic properties with PPMS (Quantum Design) magnetometry after exposure to air for 7 days. The $M_{s}$ value for the nanoparticles was 180 emu per g-Fe (Scheme 3). The obtained saturation magnetization is nearly twice as large as for commercial SPION contrast agent Resovist (95 emu per g-Fe) and close to that of bulk iron (218 emu per g-Fe) [31].

The mass fraction of α -Fe in the SiO ${}_{2}$ -coated cubic core NPs was found to be 33% by using total reflection X-ray fluorescence spectroscopy (TRXF) Picofox S2 and elemental analysis as described in the SM. This value was used to calculate the mass of iron in the NPs for MRI measurements reported in this paper (details below). The mass fraction of iron for spherical maghemite ( γ -Fe ${}_{2}$ O ${}_{3}$ @SiO ${}_{2}$ ) was found to be 27% using the same methods.

The surface of the SiO ${}_{2}$ -coated iron NPs was further modified with 3-aminopropyltriethoxy-silane (NH ${}_{2}$ -silane) for additional decoration with functional molecules, such as albumin. The NH ${}_{2}$ -silane coating was successfully implemented as confirmed with Fourier transform infrared (FTIR) spectroscopy (Scheme S1 in SM). The transverse relaxivity (r ${}_{2}$ ) of the as-synthesized cubic core α -Fe@SiO ${}_{2}$ NPs was tested with a clinical 3.0 T Philips Achieve MRI scanner. As reference compounds, commercially available spherical maghemite coated with SiO ${}_{2}$ ( γ -Fe ${}_{2}$ O ${}_{3}$ @SiO ${}_{2}$ ) was used (the latter structure is confirmed by PXRD analysis in Scheme S2). SiO ${}_{2}$ coating was implemented with the same procedure as described above. γ -Fe ${}_{2}$ O ${}_{3}$ @SiO ${}_{2}$ NPs had a core diameter of 60 nm (TEM images are shown in Scheme S2).

**Scheme 4:**
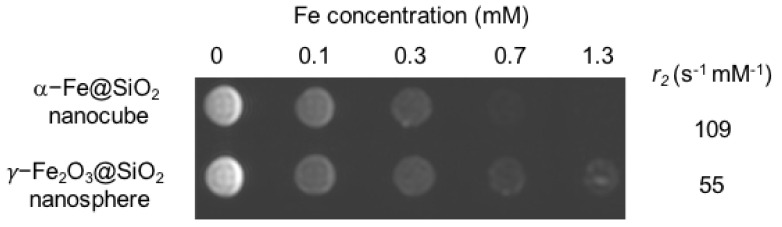
T ${}_{2}$ weighted contrast MRI image of α -Fe@SiO ${}_{2}$ nanocubes and γ -Fe ${}_{2}$ O ${}_{3}$ @SiO ${}_{2}$ nanospheres with increasing Fe concentrations and their r ${}_{2}$ values measured by 3.0 T clinical MRI system.

The obtained r ${}_{2}$ values were 55 s ${}^{-1}$ mM ${}^{-1}$ for spherical γ -Fe ${}_{2}$ O ${}_{3}$ @SiO ${}_{2}$ , and 109 s ${}^{-1}$ mM ${}^{-1}$ for cubic core α -Fe@SiO ${}_{2}$ (Scheme 4). The results show that pure metal α -Fe@SiO ${}_{2}$ NPs have nearly twice as high r ${}_{2}$ relaxivity compared to maghemite NPs. This finding can be attributed to the larger $M_{s}$ values of pure metal NPs. The literature r ${}_{2}$ values of iron oxides magnetite and maghemite vary according to particle size and the size of the polymer shell. In general, larger NPs have enhanced r ${}_{2}$ relaxivity and, depending on the study, the values for spherical SPIONs range from as little as 13 s ${}^{-1}$ mM ${}^{-1}$ to 385 s ${}^{-1}$ mM ${}^{-1}$ [11,32]. Nevertheless, in our study, α -Fe NPs showed clearly enhanced MRI relaxivity compared to maghemite NPs.

Dynamic light scattering studies revealed the average hydrodynamic size (D*h*) of NPs to be 100–200 nm for α -Fe ${}_{2}$ O ${}_{3}$ and α -Fe ${}_{2}$ O ${}_{3}$ @SiO ${}_{2}$ , 200–400 nm for α -Fe@SiO ${}_{2}$ and 600–800 nm for α -Fe@SiO ${}_{2}$ @NH ${}_{2}$ -silane in Milli-Q (MQ) water as well in the toxicity testing medium (see SM Table S1). D*h* of NPs was larger than the primary core with the SiO ${}_{2}$ shell size determined by TEM. The polydispersity index (PDI) of NPs was between 0.07 and 0.31 (SM Table S1), showing the monodispersity and stability of NP solutions. Subsequently, cytotoxicity characterization was performed on the series of synthesized NPs shown in Scheme 1. To assess the possible adverse effects of NPs on living cells, an in vitro toxicity assay with the HepG2 cell line in vitro was performed (for details see SM).

**Scheme 5:**
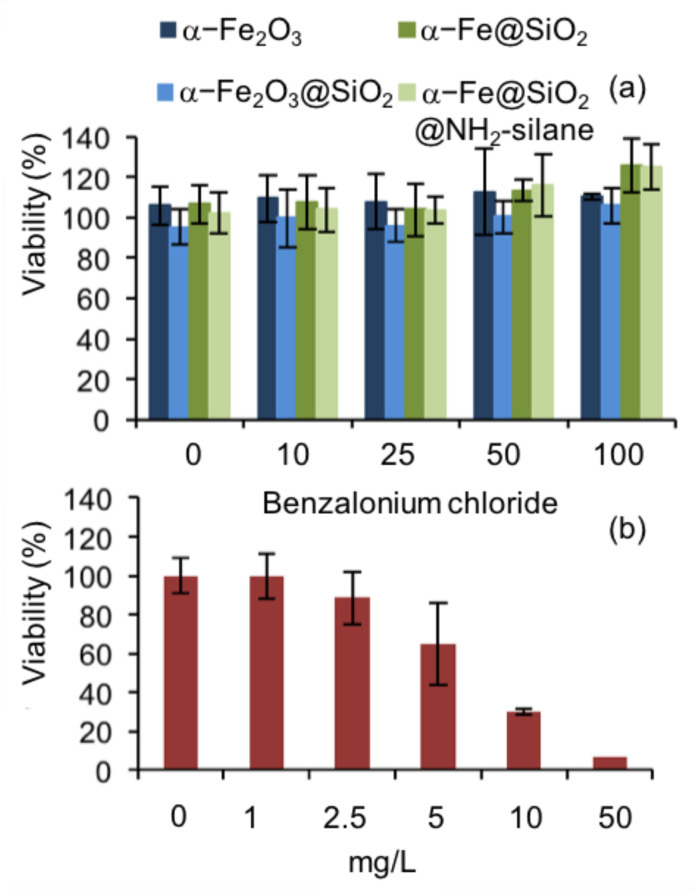
Viability of HepG2 cells in vitro determined by Resazurin assay after 24 h exposure to cubic core Fe nanoparticles (**a**) or benzalkonium chloride (**b**). Average from three independent experiments with standard deviation is shown.

HepG2, the model for human liver cells in vitro, was chosen since liver is the primary target organ of xenobiotics and is the major accumulation site for Fe-based NPs [33]. While the toxic chemical benzalkonium chloride (a chosen positive control) remarkably reduced the viability of HepG2 cells at 5 mg/L, none of the tested NPs exhibited toxicity at the highest concentration tested (100 mg/L) (Scheme 5). According to the literature, the toxicity data on the adverse effect of iron oxide NPs on human cell lines in vitro are heterogeneous and depend on the NP type and functionalization, tested cell lines and toxicity endpoint used. However, shedding of Fe ions from NP cores may induce the production of excessive ROS that may lead to cytotoxicity or/and oxidative DNA damage and genotoxicity. In the case of cytotoxicity, the mechanism of action of iron oxide NPs has been associated with ROS and resulting oxidative damage [22]. Therefore, we then assessed the possible sub-toxic adverse effects of synthetized NPs and measured the induction of abiotic ROS using H ${}_{2}$ DCFDA, a ROS-sensitive fluorescent probe (see the SM).

**Scheme 6:**
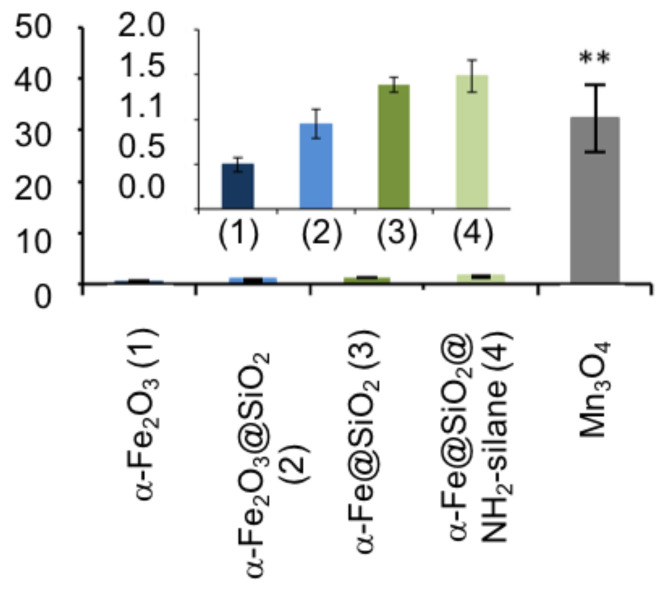
Induction of abiotic ROS with four different Fe NPs and Mn ${}_{3}$ O ${}_{4}$ NPs (a positive control) [34] measured at a concentration of 100 mg/L using 2 ${}^{\prime }$ ,7 ${}^{\prime }$ -dichlorofluorescein diacetate (H ${}_{2}$ DCFDA) assay after a 45 min incubation. Average from three independent experiments with standard deviation is shown; ** *p* < 0.01. The inset is a zoomed-in view of the synthesized NPs.

While ROS-generating Mn ${}_{3}$ O ${}_{4}$ NPs induced remarkable ROS at concentrations of 100 mg/L, none of tested Fe NPs caused the production of abiotic ROS at the highest concentration (100 mg/L) tested (Scheme 6). Therefore, the used toxicity test and abiotic ROS assay revealed that under the conditions used, the synthetized NPs showed no evidence of nanotoxicity up to a high concentration, 100 mg/L, making them promising candidates for further biocompatibility studies and biomedical applications. The upper limit value of the concentration used in the nanotoxicology research is sufficient for an MRI study.

## 3. Conclusions

In conclusion, we have synthesized cubic core α -Fe NPs with a SiO ${}_{2}$ coating and further organic functionalization with NH ${}_{2}$ -silane. The cubic core α -Fe NPs’ $M_{s}$ value of as much as 180 emu per g-Fe is quite close to that of bulk iron, 218 emu per g-Fe, and results in high MRI relaxivity r ${}_{2}$ . We have indeed determined that the cubic core α -Fe NPs’ r ${}_{2}$ value was larger than for maghemite NPs, 109 s ${}^{-1}$ mM ${}^{-1}$ compared to 55 s ${}^{-1}$ mM ${}^{-1}$ . Furthermore, cytotoxicity studies showed that the synthesized NPs were not toxic to liver cells in vitro even at 100 mg/L, revealing them to be biocompatible.

## Data Availability

The data presented in this study are available on request from the corresponding author.

## Supplementary Materials

The following supporting information can be downloaded at: https://www.mdpi.com/1996-1944/15/6/2228/s1, Scheme S1: IR spectra of α -Fe@SiO ${}_{2}$ cubic nanoparticles before (black) and after (blue) coating with NH ${}_{2}$ -silane; Scheme S2: (a) Powder XRD patterns of γ -Fe ${}_{2}$ O ${}_{3}$ @SiO ${}_{2}$ and (b) TEM image of γ -Fe ${}_{2}$ O ${}_{3}$ @SiO ${}_{2}$ ; Table S1: Summary on characterization of cubic Fe nanoparticles used in the current study.
