# Supplementary material for: Cubic Iron Core–Shell Nanoparticles Functionalized to Obtain High-Performance MRI Contrast Agents
Source: Materials (Basel). 2022 Mar 17;15(6):2228. doi: 10.3390/ma15062228 (PMC8955863; doi:10.3390/ma15062228)
Supplement: Supplementary file 1 [file materials-15-02228-s001.zip › materials-1626672-supplementary.pdf]

Supplementary materials

Cubic Iron Core-Shell Nanoparticles Functionalized to Obtain High-Performance MRI Contrast Agents

Maria Volokhova [1,2], Anna Shugai [1], Masahiko Tsujimoto [3], Anna-Liisa Kubo [1], Sven Telliskivi [4], Mait Nigul [5], Peep Uudeküll [1], Heiki Vija [1], Olesja M. Bondarenko [1], Jasper Adamson [1], Anne Kahru [1], Raivo Stern [1] and Liis Seinberg [1,*]

[1] National Institute of Chemical Physics and Biophysics, Akadeemia tee 23, 12618 Tallinn, Estonia; maria.volokhova@kbfi.ee (M.V.); anna.shugai@kbfi.ee (A.S.); anna-liisa.kubo@kbfi.ee (A.-L.K.); peep.uudekull@gmail.com (P.U.); heiki.vija@kbfi.ee (H.V.); olesja.bondarenko@kbfi.ee (O.M.B.); jasper.adamson@kbfi.ee (J.A.); anne.kahru@kbfi.ee (A.K.); raivo.stern@kbfi.ee (R.S.)

[2] Department of Chemistry and Biotechnology, Tallinn University of Technology, Akadeemia tee 15, 12618 Tallinn, Estonia

[3] Institute for Integrated Cell-Materials Sciences, Kyoto University, Yoshida-Ushinomiyacho, Sakyo-ku, Kyoto 606-8501, Japan; tsujimoto@icems.kyoto-u.ac.jp

[4] North Estonia Medical Centre, 19 J. Sütiste tee, 13419 Tallinn, Estonia; s.telliskivi@gmail.com

[5] Tartu University Hospital, Radiology Clinic, 1a L. Puusepa St., 50406 Tartu, Estonia; mait.nigul@kliinikum.ee

* Correspondence: liis.seinberg@kbfi.ee

1. Synthesis of Cubic Core Fe_2O_3 Nanoparticles

Synthesis of Fe_2O_3 will be discussed in detail in another paper by L. Seinberg et al.[1] Monodispersed cubic Fe_2O_3-NPs were synthesized by a facile one-step solvothermal route from ferric nitrite ($Fe(NO_3)_3$ $9H_2O$; 99.9%, Sigma-Aldrich Chemical Co, Saint Louis, MO, USA), *N,N*-dimethyl formamide (DMF; 99.8%, Sigma-Aldrich Chemical Co, Taufkirche, Bavaria, Germany) and poly(N-vinyl-2-pyrrolidone) (PVP, Sigma-Aldrich Chemical Co, Taufkirche, Bavaria, Germany). The reaction mixture was stirred for 30 min and sealed to an autoclave. The reaction mixture was heat controlled at 200 °C for several days. Subsequently, the reaction mixture was cooled down to room temperature, followed by three washing cycles with ethanol. The diameter of the nanocubes was approximately 40 nm.

2. Synthesis of Cubic Hematite SiO_2-Coated (Fe_2O_3@SiO_2) Nanoparticles

The SiO_2 coating was performed using a method previously published by Yamamoto et al. [2]. The SiO_2 coating was carried out using tetraethyl orthosilicate (TEOS, 98%, Sigma-Aldrich Chemical Co Taufkirche, Bavaria, Germany), where 1 mL of TEOS was added to 30 mL ethanol solution and stirred for 1 h. The Fe_2O_3–NPs (109 mg/g) in ethanol solution were mixed with ethanol-water solution (1:10), to which 2.5 mL of ammonium hydroxide (NH_3OH, 28%, Sigma-Aldrich Chemical Co, Taufkirche, Bavaria, Germany) was added. The reaction mixture was sealed, stirred and continuously sonicated at room temperature for one hour. TEOS-ethanol was added to Fe_2O_3 NPs reaction mixture in the course of 8 h. Using magnet NPs were extracted from the reaction mixture and were washed with ethanol and dried in air to obtain Fe_2O_3@SiO_2 powder (Scheme S2).

3. CaH_2 Reduction Reaction to Obtain Cubic Fe@SiO_2 Nanoparticles.

The reduction reaction with reducing agent, CaH_2 was carried out according to the method previously described elsewhere by Yamamoto et al. There were some modifications of the weight excess and reaction temperatures. α-Fe_2O_3@SiO_2 sample was finely ground with three weight excess of CaH_2 (99.6%, Sigma-Aldrich Chemical Co, Taufkirche, Bavaria, Germany) in an Ar-filled glove box, sealed in an evacuated Pyrex tube and heated at 260 °C for 96 h. By-products, such as CaO and residual CaH_2, were removed from the reaction mixture by washing it with NH_4Cl/methanol (99.9%, Sigma-Aldrich Chemical Co, Taufkirche, Bavaria, Germany) solution in air.

4. Fe Content Estimation in the SiO2 Coated Nanoparticles Using TRXF:

Determining the Fe content in the NPs was important to obtain saturation magnetization values in the units of $m^3 \cdot kg^{-1}$—Fe. The magnetization as obtained from Physical Property Measurement System (PPMS-14T, Quantum Design, San Diego, CA, USA) was divided by the mass of pure iron in the sample. The latter was determined by TRXF and atomic absorption spectroscopy with both of these methods giving very similar results. In order to estimate the amount of Fe in the α-Fe@SiO_2 nanocubes, total reflection X-ray fluorescence spectroscopy (TRXF) Picofox S2 (Bruker AXS Microanalysis GmbH, Berlin, Germany) was carried out. NP suspension was mixed 1:1 with gallium internal standard and 5 μL of the as-prepared mixture was pipetted onto a quartz carrier disc (Bruker, Berlin, Germany). The concentration of Fe was quantified with Spectra software (Bruker, AXS Microanalysis GmbH, Berlin, Germany). Iron content in the cubic α-Fe@SiO_2 NPs sample was measured to be 33% wt and that of spherical maghemite (γ-Fe_2O_3@SiO_2) 27% wt.

5. Atomic Absorption Spectroscopy Instrumentation

In addition, Spectra AA 220F flame atomic absorption spectrometer (Varian, Mulgrave, Victoria, Australia) equipped with deuterium lamp for background correction was used. Acetylene of 99.99% purity (AGA, Helsinki, Finland) was used as the fuel gas. Iron was extracted from the samples with concentrated nitric and hydrofluoric acids (1 mL of the mixture 1:1) in the water bath at 85 °C for 120 min. After cooling down the samples were diluted to 100 mL with Milli-Q water. Iron content in the cubic α-Fe@SiO_2 NPs was 33% wt and that of spherical maghemite (γ-Fe_2O_3@SiO_2) 27% wt.

6. Coating with 3-aminopropyltrimethoxysilane (NH_2-silane)

Silane coating was carried out using 3-aminopropyltrimethoxysilane **(**NH_2-silane), (97%, Sigma-Aldrich Chemical Co, Taufkirche, Bavaria, Germany) to obtain Fe@SiO_2@NH_2-silane NPs. 1 mL of NH_2-silane was added to 30 mL ethanol solution and stirred for 1 h. The Fe@SiO_2 NPs in ethanol were mixed with ethanol-water solution ratio (1:10), followed by the addition of 2.5 mL of NH_3OH (28%, Sigma-Aldrich Chemical Co, Taufkirche, Bavaria, Germany) and stirred for 1 h. Subsequently, silane-ethanol solution was added to Fe@SiO_2 NPs during 8 h, while stirring and sonicating the mixture.

7. Infrared Spectroscopy (IR)

IR measurements were made with an interferometer Vertex 80v Bruker FT/IR, Berlin, Germany with Glowbar (resistively heated SiC rod) as a light source and a Liquid Nitrogen cooling - Mercury Cadmium Telluride detector (LN-MCT). Measurements were made at room temperature (298 K), using 2 mm aperture and 0.5 cm^{-1} resolution. IR spectra were acquired on a pressed (60 MPa pressure) pellet (diameter 3 mm) of a sample material mixed with pure and dry KBr powder (Spectra shown in Scheme S1). Such dilution was needed because otherwise the absorption lines were too strong. During the measurement, sample was in evacuator till 1hPa (1×10^{-3}, atm) pressure compartment.

8. Reagents

Nitric acid (65%) and hydrofluoric acid (40%) (Sigma-Aldrich, Taufkirche, Bavaria, Germany) were used for sample digestion. The stock atomic spectroscopy standard solution (1000 mg/L) of Fe was purchased from MERCK, Buchs, Switzerland and used for calibration of equipment. Multielement Quality Control Standard 26 (High-purity standards, Charleston, SC, USA) was gradually diluted with 4% nitric acid solution and used for quality control. The deionized water (Milli-Q, Millipore, Burlington, MA, USA) was used for the preparation of all solutions.

9. Materials Characterization

Powder x-ray diffraction measurements were carried out using Panalytic Powder 3 with Cu K_α radiation (λ = 0,154 nm) beam voltage 30 kV and beam current 40 mA. Patterns

were collected in a range of 20 ° to 90 ° with the step of 0.02 °and the exposure time of 2 s. TEM (JEOL JEM-1400) low and high-magnetification observation was used to characterize obtained nano cubes morphology. TEM specimens were prepared by dropping a nanoparticle solution on a copper grid and air dried. In order to study the magnetic properties were measured by a Physical Property Measurement System (Quantum Design PPMS-14T, San Diego, CA, Germany) with a vibrating sample magnetometer (VSM, Quantum Design San Diego, CA, Germany) attachment. IR spectra were collected on Bruker FT/IR, Berlin, Germany. The samples were mixed with KBr and compressed into pellets. The NP samples were scanned by clinical whole-body MRI system Achieva 3.0T, Philips, Horgen Switzerland, The Netherlands. The relaxivity r_2 was calculated from signal intensities acquired by multi-echo TSE sequence with the following parameters: repetition time TR = 2000 ms, echo train lentgh ETL (number of echoes) = 8, echo time TE = 10 to 80 ms with increment 10 ms, flip angle FA=90 °, FOV=160 mm, image matrix 512 x 512, slice thickness 5 mm, number of excitations NEX = 2.

10. Evaluation of the Toxicity of NPs to HepG2 Cells in vitro

The toxicity of NPs in vitro was assessed using immortalised hepatocellular carcinoma cell line HepG2 (ATCC HB-8065, Manassas, VA, United States). Cells were cultured in DMEM Dulbecco's Modified Eagle Medium with GlutaMax Thermo Fisher Scientific, Helsinki, Finland, 1 g/L D-glucose and pyruvate (Gibco) supplemented with 10% of non-heat activated FBS (Gibco), 100 U/mL penicillin (Naxo) and 100 U/mL Streptomycin/Penicillin (Naxo). Before the toxicity test, 200 μL of cells were seeded in 96-well plates (Cellstar, Greiner) at density 10^4 cells per well and incubated for 24 h (37 °C, 95% humidity and 5% CO_2). After 24 h cells were exposed to 100 μL of 0 (blank — DMEM medium); 3; 6; 12.5; 25; 50 and 100 mg/L Fe NPs diluted in DMEM medium and incubated for 24 h at 37 °C, 95% humidity and 5% CO_2. Benzalkonium chloride was used as a positive control and abiotic NPs were used to assess possible interference of NPs with the assay components. After 24-h exposure, the supernatants were aspirated, cells were washed with 100 μL PBS and cell viability was measured using Resazurin assay (0.03 mg/mL of resazurin in cell culture medium, incubated with cells for 2 h at 37 °C). The fluorescence (excitation at 530 nm/emission at 570 nm) was measured using Fluoroskan Ascent FL (Thermo Labsystems, Philadelphia, PA, United States). Percentage of viable cells was calculated by dividing the fluorescence values measured in NP suspensions or positive controls by blank (DMEM medium with dye). Test was performed three times in triplicates.

11. Evaluation of the abiotic generation of reactive oxygen species

The potential of NPs to generate abiotic reactive oxygen species (ROS—Table S1) was performed as described by Aruoja et al [3]. Briefly, fluorescent probe 2,7-Dichlorodihydrofluorescein diacetate H_2DCF-DA was dissolved in ethanol at concentration 5.13 mM and stored in the dark at −20 °C. Before use H_2DCF-DA was deacetylated to 2,7-Dichlorodihydrofluorescein (H_2DCF) with 0,01 M NaOH for 30 min in the dark and diluted in PBS to 52 μM. The H_2DCFsolution was kept on ice, protected from light and used within 1 h after dilution. For the test, 100 μL of NP suspensions at final concentration of 3; 6; 12.5; 25; 50 and 100 mg/L were pipetted onto the wells of 96-well black microplate and 100 μL of 52 μM H_2DCF solution in PBS was added to NPs. Mn_3O_4 NPs (15.2 nm primary size) served as a positive control by Aruoja et al [3]. Mixtures were incubated at room temperature for 45 min. The fluorescence (excitation at 485 nm/emission at 527 nm) was measured using Fluoroskan Ascent FL (Thermo Labsystems, Philadelphia, PA, United States). The abiotic ROS was calculated in relative fluorescence units (RFU) by dividing the fluorescence values of H_2DCF in NP solutions by the values in blank (distilled water with dye).

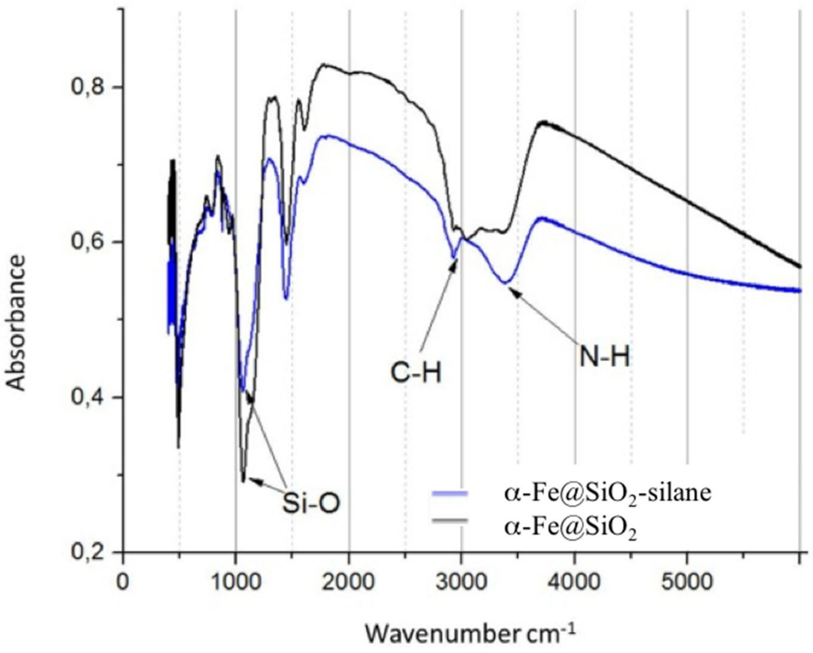

Scheme S1. IR spectra of α-Fe@SiO2 cubic nanoparticles before (black) and after (blue) coating with NH_2-silane.

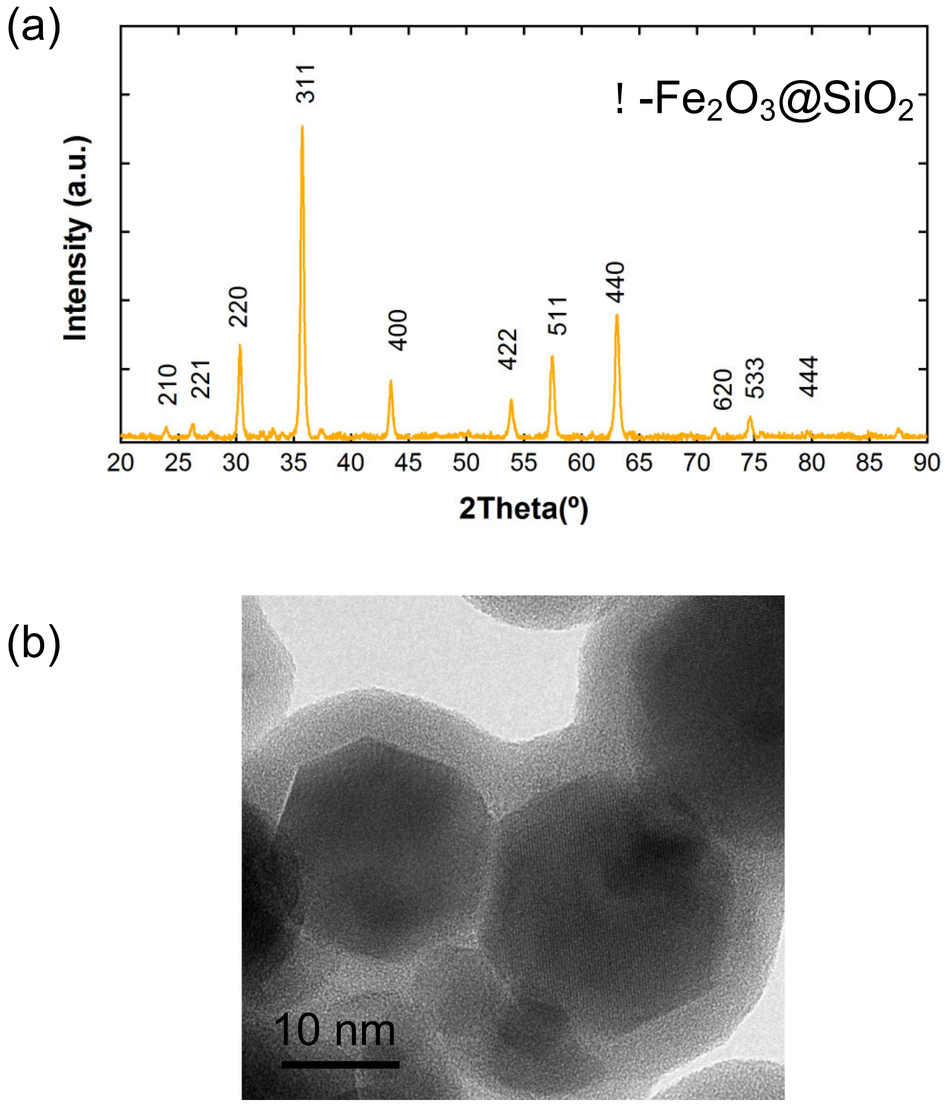

Scheme S2. (a) Powder XRD patterns of γ-Fe_2O_3@SiO_2 and (b) TEM image of γ-Fe_2O_3@SiO_2.

Table S1. Summary on characterization of cubic Fe nanoparticles used in the current study.

Characteristic	α-Fe_2O_3	α-Fe_2O_3-SiO_2	α-Fe-SiO_2	α-Fe-SiO_2-silane
Primary size (nm)	40	50	50	50
Shape	cubic	cubic	quasi-cubic	quasi-cubic
Coating SiO_2 (%)	None	Silica	Silica	Silica-silane
Hydrodynamic size in milliQ water; MQ[a] (nm)	120 ± 1.2	179 ± 35	236 ± 34	728 ± 239
Polydispersity index in MQ[a]	0.07 ± 0.03	0.24 ± 0.0	0.09 ± 0.01	0.31 ± 0.02
Hydrodynamic size in toxicity test medium[a] (nm)	112 ± 0.5	196 ± 8	355 ± 38	644 ± 186
Abiotic generation of reactive oxygen species (ROS)[b]	No	No	No	No

[a] Measured by dynamic light scattering at concentration of NPs 200 mg/L using Malvern Zetasizer Nano-ZS (Malvern Instruments, Malvern, United Kingdom).

[b] Reactive oxygen species (ROS) were determined using 2′,7′-dichlorofluorescin diacetate and using Mn_3O_4 NPs (primary size 15.2 nm) as a positive control as described in Aruoja et al. (2015).

References

1. Seinberg, L.; Volokhova, M. Metal-Based Core Nanoparticles, Synthesis and Use. WO2021/144006A1, 22 July 2021.
2. Matsumoto, A.; Sugiura, T.; Kobashi, M.; Yamamoto, S. Preparation and Magnetic Property of Nano-sized Iron Powder Particles Covered with Silica Film by Calcium Hydride Reduction Method. *J. Jpn. Soc. Powder Powder Met.* **2019**, *66*, 429–433, https://doi.org/10.2497/jjspm.66.429.
3. Aruoja, V.; Pokhrel, S.; Sihtmäe, M.; Mortimer, M.; Mädler, L.; Kahru, A. Toxicity of 12 metal-based nanoparticles to algae, bacteria and protozoa. *Environ. Sci. Nano* **2015**, *2*, 630–644, https://doi.org/10.1039/c5en00057b.
